# A D-cysteine desulfhydrase, SlDCD2, participates in tomato fruit ripening by modulating ROS homoeostasis and ethylene biosynthesis

**DOI:** 10.1093/hr/uhad014

**Published:** 2023-02-01

**Authors:** Yu-Qi Zhao, Kang-Di Hu, Gai-Fang Yao, Si-Yue Wang, Xiang-Jun Peng, Hua Zhang

**Affiliations:** School of Food and Biological Engineering, Hefei University of Technology, Hefei 230009, China; School of Food and Biological Engineering, Hefei University of Technology, Hefei 230009, China; School of Food and Biological Engineering, Hefei University of Technology, Hefei 230009, China; School of Food and Biological Engineering, Hefei University of Technology, Hefei 230009, China; School of Food and Biological Engineering, Hefei University of Technology, Hefei 230009, China; School of Food and Biological Engineering, Hefei University of Technology, Hefei 230009, China

## Abstract

Hydrogen sulfide (H_2_S) is involved in multiple processes during plant growth and development. D-cysteine desulfhydrase (DCD) can produce H_2_S with D-cysteine as the substrate; however, the potential developmental roles of DCD have not been explored during the tomato lifecycle. In the present study, *SlDCD2* showed increasing expression during fruit ripening. Compared with the control fruits, the silencing of *SlDCD2* by pTRV2-*SlDCD2* accelerated fruit ripening. A *SlDCD2* gene-edited mutant was constructed by CRISPR/Cas9 transformation, and the mutant exhibited accelerated fruit ripening, decreased H_2_S release, higher total cysteine and ethylene contents, enhanced chlorophyll degradation and increased carotenoid accumulation. Additionally, the expression of multiple ripening-related genes, including *NYC1*, *PAO*, *SGR1*, *PDS*, *PSY1*, *ACO1*, *ACS2, E4, CEL2*, and *EXP* was enhanced during the *dcd2* mutant tomato fruit ripening. Compared with the wild-type fruits, *SlDCD2* mutation induced H_2_O_2_ and malondialdehyde (MDA) accumulation in fruits, which led to an imbalance in reactive oxygen species (ROS) metabolism. A correlation analysis indicated that H_2_O_2_ content was strongly positively correlated with carotenoids content, ethylene content and ripening-related gene expression and negatively correlated with the chlorophyll content. Additionally, the *dcd2* mutant showed earlier leaf senescence, which may be due to disturbed ROS homeostasis. In short, our findings show that *SlDCD2* is involved in H_2_S generation and that the reduction in endogenous H_2_S production in the *dcd2* mutant causes accelerated fruit ripening and premature leaf senescence. Additionally, decreased H_2_S in the *dcd2* mutant causes excessive H_2_O_2_ accumulation and increased ethylene release, suggesting a role of H_2_S and *SlDCD2* in modulating ROS homeostasis and ethylene biosynthesis.

## Introduction

Hydrogen sulfide (H_2_S) is emerging as a signaling molecule in all living organisms. Accumulating evidence over the past decades has shown that H_2_S participates in a vast number of physiological processes critical for plant growth and development, such as stomatal movement, flowering, and responses to abiotic stresses [1–4]. Furthermore, multiple studies have revealed that H_2_S can delay leaf and flower senescence and prevent the ripening of fruits, such as strawberry, tomato, and kiwifruit, by modulating the antioxidant system and antagonizing the ethylene pathway [5–9]. For example, H_2_S could maintain the freshness of cut flowers by reducing the oxidative damage caused by excessive reactive oxygen species (ROS). Hu *et al.* [10] revealed that the senescence of mulberry fruit is delayed by exogenous H_2_S treatment and that the mechanism is related to the enhanced activity of antioxidant enzymes. Additionally, H_2_S counteracts the biological effect of ethylene and thereby alleviates the ripening and senescence of banana and tomato during postharvest storage [7, 11]. Previous research has mainly revealed the mechanism through which exogenous H_2_S prevents fruit ripening, but the influence of endogenous H_2_S in fruit ripening still needs further investigation.

In higher plants, H_2_S emission has long been observed in pumpkin, cucumber, cantaloupe, corn, and others [12]. Currently, H_2_S can be generated during sulfur assimilation and cysteine decomposition. In the sulfate assimilation pathway, H_2_S is formed mainly through sulfite reductase (SiR), and sulfide is then integrated into the first organic sulfur-containing molecule cysteine by O-acetylserine thiol lyase (OAS-TL) [13]. Via another route, H_2_S is generated from L-cysteine by the catalysis of L-cysteine desulfhydrase (LCD) or from D-cysteine by D-cysteine desulfhydrase (DCD), but the two enzymes are not structurally related and are evolutionarily independent. DCD catalyzes the production of H_2_S from D-cysteine, which was first reported in *Escherichia coli* [14]. Two putative DCD homologous genes were later found in Arabidopsis, and these are called AtDCD1 (At1g48420) and AtDCD2 (At3g26115). DES1, a member of the OAS-TL family, is regarded as the main cysteine desulfhydrase in the cytosol of Arabidopsis cells [15]. In addition, β-cyanoalanine synthase (CAS) produces H_2_S during the detoxification of cyanide [16]. The common characteristic of these cysteine desulfhydrases is that they all use pyridoxal 5′-phosphate (PLP) as the cofactor. The mutation of DES1 or SiR as an endogenous H_2_S-generating enzyme results in early leaf senescence, which suggests that endogenous H_2_S may be a signal that influences the senescence process of plants [15, 17, 18].

Cysteine plays a critical role in plant primary and secondary metabolism through its incorporation into proteins and integration into sulfur-containing defense compounds [19]. L-cysteine, not D-cysteine, is the amino acid stereoisomer that is incorporated into peptides. D-amino acids have come to be regarded as physiological signaling molecules in mammals, particularly in the central nervous system [20]. For instance, endogenous D-cysteine in the mammalian brain acts as a negative impact factor of growth factor signaling during cortical development [20]. In plants, D-cysteine can be assembled into camalexin, which confers toxicity to several species of fungi [21]. DCD activity can be detected in multiple plants, such as *Arabidopsis*, *Spinacia oleracea*, *Chlorella fusca*, and *Nicotiana tabacum*, which suggests the presence of D-cysteine in plants. A serine/aspartate racemase has been identified as being involved in the generation of D-serine/aspartate in plants [22], but the potential for a cysteine racemase in plants has not yet been reported. The cysteine concentrations in the cytosol, which is the major site of cysteine synthesis, are estimated to be greater than 300 μM, whereas other cell compartments, such as the chloroplast and mitochondrion, each contain less than 10 μM cysteine [23]. The concentrations of free D-amino acids such as D-Asp, D-Asn, D-Glu, and D-Gln are approximately 0.2% to 8% relative to the corresponding L-amino acids in plants; however, cysteine data were not provided [24]. Therefore, research on D-amino acids or their related metabolism in plants is an emerging field with many open questions.

In Arabidopsis, Cd-induced WRKY13 promotes the production of H_2_S by binding to the *DCD* (gene encoding DCD) promoter and thereby enhances plant tolerance to Cd [25]. In our previous study, we found that a *SlLCD1* gene-edited mutant displays premature fruit ripening, which suggests that *SlLCD1* is a negative impact factor of fruit ripening [26]. The role of *SlLCD1* in natural and dark-induced leaf senescence was thus studied, and the results showed that *SlLCD1* mutation causes earlier leaf senescence [9]. However, whether DCD participates in tomato fruit ripening and leaf senescence remains unclear.

Tomato is a typical climacteric fruit and an economically important vegetable worldwide [27]. Fruit ripening is associated with changes in metabolic pathways. Pigments, particularly chlorophyll and carotenoids, show substantial changes during tomato fruit ripening that lead to the change in fruit color from green to red [28]. In the ripening process, the degradation of chlorophyll begins with the conversion of chlorophyll b to chlorophyll a [29]. PAO (pheophorbide a monooxygenase) and PPH (pheophytin pheophorbide hydrolase) are then needed for subsequent chlorophyll degradation. In addition, SGR (STAY-GREEN) proteins can affect the degradation of chlorophyll by interacting with chlorophyll-degrading enzymes, and SGR1 in tomato can promote chlorophyll degradation. Tomato fruit ripening is alco accompanied by carotenoids biosynthesis; during this process, phytoene synthase (PSY) is the key rate-limiting enzyme, and phytoene dehydrogenase (PDS) is involved in subsequent steps leading to the formation of lycopene. Among plant hormones, ethylene elicits profound metabolic changes during tomato fruit ripening. During the ripening process, the ACC synthase- and ACC oxidase-encoding genes *ACS1*, *ACS2*, *ACS4*, *ACO1*, and *ACO3* show increasing expression, which suggests their important roles in tomato fruit ripening. In a biological context, ROS, which are byproducts of natural aerobic metabolism, are composed of superoxide anion (O_2_^•−^), hydrogen peroxide (H_2_O_2_), and hydroxyl radicals (·OH), which have been previously associated with oxidative stress. However, accumulating evidence has revealed that ROS also operate as intracellular signaling molecules that participate in multiple physiological processes [30]. During peach fruit ripening, O_2_^•−^ is required at the middle stage of fruit development, and H_2_O_2_ acts as a potential signaling molecule to stimulate the late stage of fruit development [31]. In addition, H_2_O_2_ is a signaling molecule that accumulates during the ripening of nectarine fruit and at the beginning of grape berry ripening [32, 33]. Oxidative stress is also an integrative factor for triggering tomato fruit ripening, which suggests the signaling role of oxidants in initiating fruit ripening [34].

The interaction of H_2_S with other signals such as ethylene and ROS has attracted increasing attention. H_2_S alleviates oxidative stress by dynamically modulating antioxidant enzyme systems [35, 36] and antagonizing the effect of ethylene; however, the mechanism through which endogenous H_2_S affect ROS homeostasis and ethylene biosynthesis during fruit ripening is largely unclear. Thus, in the present work, the function of *SlDCD2* in tomato fruit ripening was investigated by virus-induced gene silencing (VIGS) and CRISPR/Cas9-mediated gene editing. In addition, the effect of *SlDCD2* mutation on ROS homeostasis and ethylene biosynthesis was studied to reveal the role of *SlDCD2* and H_2_S in modulating ROS and ethylene metabolism.

## Results

### Phylogenetic analysis of DCDs and the expression profile and enzymatic activity of SlDCD2

**Figure 1:**
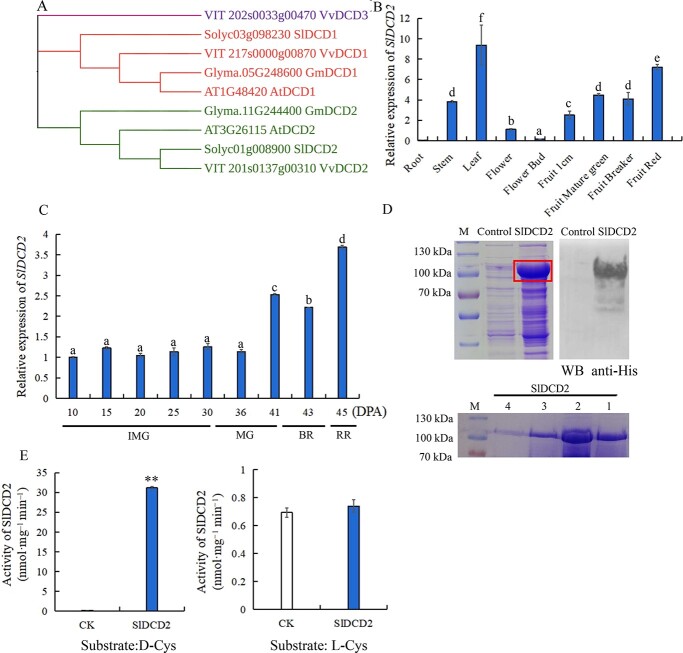
Phylogenetic tree analysis of DCD proteins in plants, tissue-specific expression of *SlDCD2*, expression level of *SlDCD2* in fruits at different ripening stages, and assay of enzymatic activity of SlDCD2 in tomato. **A** DCD proteins from *Solanum lycopersicum*, *Arabidopsis thaliana*, *Vitis vinifera*, and *Glycine max* were analysed in the phylogenetic tree. **B***SlDCD2* expression patterns in different tissues of tomato. **C** Expression level of *SlDCD2* during tomato fruit ripening at 10, 15, 20, 25, 30, 36, 41, 43, 45 DPA. BR: breaker stage; IMG: immature green stage; MG: mature green stage; RR: red ripe stage. **D** Coomassie Blue-stained SDS–PAGE and Western blot analysis of the SlDCD2 protein. The *SlDCD2* coding sequence was cloned into pCOLD, and protein expression in DE3 cells was induced by IPTG. SlDCD2 protein was then detected by Western blot using anti-His antibody. SlDCD2 protein was purified by nickel affinity chromatography using an Invitrogen Ni-NTA purification system. Lanes 1–4 present different imidazole elution fractions. The empty pCOLD vector was used as a negative control. M: protein molecular-weight markers in kDa. **E** Enzymatic activity of SlDCD2 in the presence of the substrate D/L-cysteine. The values are the means of three replicates ± their SDs based on the protein content. ^**^P < 0.01 as determined by Student’s *t* test. Significant differences at the level of *P* < 0.05 are indicated with letters.

To investigate the phylogenetic relationships between DCD proteins in tomato and other plant species, the gene encoding AtDCD1 (AT1G48420) in Arabidopsis was searched in the Phytozome v13 database, and the homologous proteins in *Solanum lycopersicum*, *Arabidopsis thaliana*, *Vitis vinifera*, and *Glycine max* were searched using the AtDCD1 protein sequence as the query. As shown in the phylogenetic tree in Fig. 1A, the identified DCDs could be classified into three groups. Subfamily I contained VvDCD1, SlDCD1, AtDCD1, and GmDCD1, and subfamily II contained VvDCD2, SlDCD2, AtDCD2, GmDCD2, and VvDCD3 and formed a single branch. The results indicated that SlDCD1/2 showed high homology with homologs in grapevine.

To explore the expression pattern of *SlDCD2* in different tomato tissues, RNA from tomato roots, stems, leaves, flowers, flower buds, 1-cm fruits, mature green fruits, breaker fruits, and red fruits was reverse-transcribed into cDNA for RT–qPCR. As shown in Fig. 1B, the expression of *SlDCD2* was relatively high in tomato leaves and increased gradually during fruit ripening, revealing the potential role of *SlDCD*2 in fruit ripening. To further investigate the potential role of *SlDCD2* in fruit ripening, we determined the expression level of *SlDCD2* in wild-type (WT) fruits at different ripening stages. The results showed that the relative expression of *SlDCD2* increased significantly during fruit ripening, particularly at 41 DPA, 43 DPA, and 45 DPA (Fig. 1C). Therefore, *SlDCD2* may play a more important role at the stage of fruit ripening.

To confirm the enzymatic activity of SlDCD2 in decomposing D-cysteine, we cloned the coding sequence of *SlDCD2* into the pCOLD vector to express the N-terminal his-tagged SlDCD2 recombinant protein. SDS–PAGE and Western blot analyses indicated that the recombinant protein was highly expressed (Fig. 1D). Subsequently, the SlDCD2-His protein was purified by Ni-affinity chromatography (Fig. 1D). Using D-cysteine as the substrate, the enzymatic activity of SlDCD2 was evaluated based on the H_2_S production rate. The enzymatic activity of the recombinant protein SlDCD2 was as high as 31.19 nmol^∙^mg^−1^ (protein)^∙^min^−1^. We also used L-cysteine as a control to show the specificity of SlDCD2 enzyme activity. The enzyme activity observed with L-cysteine as the substrate was only 0.78 nmol^∙^mg^−1^ (protein)^∙^min^−1^, which was not significantly different from that of the control group, and these findings confirmed the ability of SlDCD2 protein to catalyze the formation of H_2_S with D-cysteine and not L-cysteine as the substrate (Fig. 1E).

### The transient silencing of *SlDCD2* promotes tomato fruit ripening

**Figure 2:**
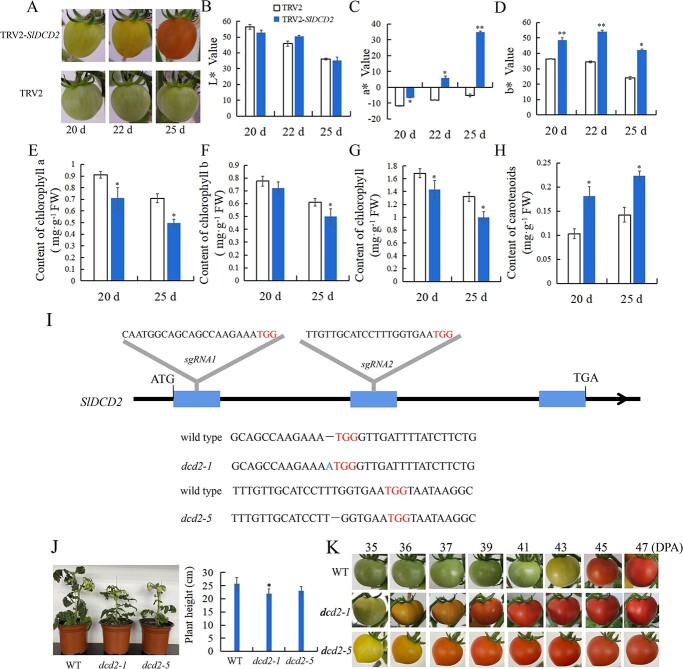
VIGS of *SlDCD2* and *dcd2* deletion lines display accelerated tomato fruit ripening. **A** Phenotype of *SlDCD2*-silenced tomato fruits. TRV2: empty TRV vector-infected fruit. TRV2*-SlDCD2*: TRV2-*SlDCD2*-infected fruit. The images were photographed at 20, 22, and 25 days after *Agrobacterium* infection. **B** The L^*^ value reflects the lightness of the fruits in **A**. **C** The a^*^ value reflects the chromaticity on a green (−) to red (+) axis. **D** The b^*^ value is the chromaticity on a blue (−) to yellow (+) axis. Changes in the contents of (**E**) chlorophyll a, (**F**) chlorophyll b, (**G**) total chlorophyll, and (**H**) carotenoids in control and *SlDCD2*-silenced tomato fruits at 20 and 25 days after infection with *Agrobacterium tumefaciens*. **I** Generation of *dcd2* tomato lines by CRISPR/Cas9. The protospacer-adjacent motif (PAM) is indicated in red. The dashes indicate deletions and insertions of bases. **J** Overall phenotype and plant height of the two mutant lines *dcd2–1* and *dcd2–5* after 60 days of growth. **K** The two mutant lines, *dcd2–1* and *dcd2–5*, display accelerated fruit ripening, as revealed by fruits photographed at 35, 36, 37, 39, 41, 43, 45, and 47 days post anthesis (DPA). The data are expressed as the means ± SDs of three replicates. ^**^P < 0.01; ^*^P < 0.05.

The vector tobacco rattle virus (TRV)-*SlDCD2* was used to transiently silence the expression of *SlDCD2*. As shown in Fig. 2A, after infection, *SlDCD2*-silenced fruit displayed yellow coloration on Day 22 and turned red on Day 25, whereas the control fruit was still green at Day 25. Chromaticity data showed that the fruit infected with TRV-*SlDCD2* exhibited higher a* and b* values compared with the control fruit (Fig. 2C and D), whereas no significant changes in lightness (L) were found (Fig. 2B).

Tomato fruit ripening is accompanied by breakdown of the thylakoid structure, loss of photosynthetic capacity of the chloroplast, and carotenoids accumulation in the chromoplast of fruit cells. Thus, the contents of chlorophyll and carotenoids in *SlDCD2-*silenced fruit were measured. Compared with the fruits of the control group, the *SlDCD2*-silenced fruits showed chlorophyll contents that were decreased by 16% and carotenoid contents that were increased by 74% at 20 days after infection. After 25 days of infection, the chlorophyll content in the *SlDCD2*-silenced fruit was decreased by 23%, and the carotenoids content was increased by 57%. Overall, the results showed that the contents of chlorophyll a (Fig. 2E), chlorophyll b (Fig. 2F), and total chlorophyll (Fig. 2G) in *SlDCD2*-silenced fruits were lower than those in control fruit, whereas the content of carotenoids (Fig. 2H) was higher in *SlDCD2*-silenced fruit than in control tomato. In summary, the silencing of *SlDCD2* accelerated chlorophyll degradation and carotenoids accumulation in the fruits.

The expression of *SlDCD2* and ripening-associated genes was analyzed by RT–qPCR. The results shown in Fig. S1A (see online supplementary material) indicate that the expression of *SlDCD2* in the *SlDCD2*-silenced fruit infected for 20 or 25 days was approximately 36% of that in the control fruit, suggesting that *SlDCD2* was successfully silenced. To further verify the effect of *SlDCD2* silencing on fruit ripening, we measured the expression of genes associated with fruit ripening and senescence, including the chlorophyll degradation genes *NYC1*, *PAO*, and *SGR1*, the carotenoids synthesis genes *PSY1* and *PDS*, the ethylene synthesis genes *ACO1* and *ACS2*, the ethylene response gene *E4*, and the cell wall metabolism gene *CEL2*, in the control and *SlDCD2*-silenced tomatoes. In *SlDCD2*-silenced fruits infected for 25 days, the expression of *NYC1* (Fig. S1B, see online supplementary material), *PAO* (Fig. S1C, see online supplementary material), and *SGR1* (Fig. S1D, see online supplementary material) was 1–1.5 times that in the control group, the expression of *PSY1* (Fig. S2A, see online supplementary material) and *PDS* (Fig. S2B, see online supplementary material) was 3- and 9-fold higher, respectively. In SlDCD2-silenced fruits infected for 20 days, compared with that in control group, the expression of *ACO1* (Fig. S2C, see online supplementary material), *ACS2* (Fig. S2D, see online supplementary material), and *E4* (Fig. S2E, see online supplementary material) was 1.1-, 20- and 10-fold that in the control fruits, respectively, and the expression of *CEL2* (Fig. S2F, see online supplementary material) was 125 times that in the control group. Therefore, the results indicated that *SlDCD2* silencing accelerated fruit ripening by improving the expression of ripening-associated genes and that *SlDCD2* positively delayed the ripening process of tomato fruit.

### Construction of an *SlDCD2* gene-edited plant by CRISPR/Cas9

Two sgRNA targets of *SlDCD2* were integrated into the CRISPR/Cas9 vector, which was further transformed into the cultivar ‘Micro Tom’ by Agrobacterium-mediated transformation. For the genotyping of positive T2 plants, the gene fragments flanking both sgRNA targets of *SlDCD2* were amplified from genomic DNA of the *dcd2* mutants. In addition, *SlDCD2* CDS amplification products with cDNA from transformed tomato lines as the template were sequenced to verify that the CDS of *SlDCD2* was mutated. Analysis of the plant genome (Fig. 2I) and CDS sequencing (Fig. S3A, see online supplementary material) showed that the ‘A’ insertion in *dcd2-1* near the first PAM caused a severe frame shift mutation, which led to a 30-amino-acid truncation mutant, and the amino acid sequence was significantly changed (Fig. S3B, see online supplementary material). A ‘T' deletion in *sgRNA2* of the *dcd2-5* CDS (Fig. 2I; Fig.S3A, see online supplementary material) led to a 119-amino-acid truncation mutant and destroyed the functional domain of SlDCD2 (Fig. 2I; Fig.S3B, see online supplementary material).

As shown in Fig. 2J, the height of the WT was approximately 26 cm, whereas the height of the *dcd2-1* mutant was only 22 cm, and that of the *dcd2-5* mutant was approximately 23 cm, suggesting a minor effect of *SlDCD2* deletion on plant growth. To further verify the mechanism through which *SlDCD2* participates in fruit ripening, the ripening process in the WT and *dcd2* fruits was monitored, and *dcd2-1* and *dcd2-5* mutants showed a similar early ripening phenotype. As shown in Fig. 2K, *dcd2-1* and *dcd2-5* fruits entered the breaker stage at 35 days post anthesis (DPA) and turned red at 39 DPA. However, the WT fruits began to turn yellow at 43 DPA and became completely red at 47 DPA. Based on the above-described result, the *dcd2-1* and *dcd2-5* fruits ripened 8 days earlier than the WT fruits, suggesting that the mutation of *SlDCD2* accelerated the ripening of tomato fruit and that *SlDCD2* delayed the ripening process of tomato fruit.

D-cysteine is degraded by DCD2 to produce H_2_S, ammonia, and pyruvate. To rule out the effect of pyruvate and ammonia, the contents of which may be slightly reduced in *dcd2* deletion mutants, WT tomato fruits at the white mature stage were soaked with H_2_O, 50 μM NaHS solution (H_2_S treatment group), 50 μM pyruvate solution, or 50 μM ammonium chloride solution for 8 h. The results presented in Fig. S4 (see online supplementary material) show that the fruits in the H_2_S group exhibited delayed fruit ripening compared with those in the H_2_O control group. Moreover, pyruvate and ammonium chloride treatment did not delay fruit ripening in comparison to the control, suggesting that H_2_S is the signal that delays fruit ripening and that the acceleration of fruit ripening in the *dcd2* deletion mutant could be attributed to a decreased H_2_S content instead of the effects of pyruvate and ammonia.

**Figure 3:**
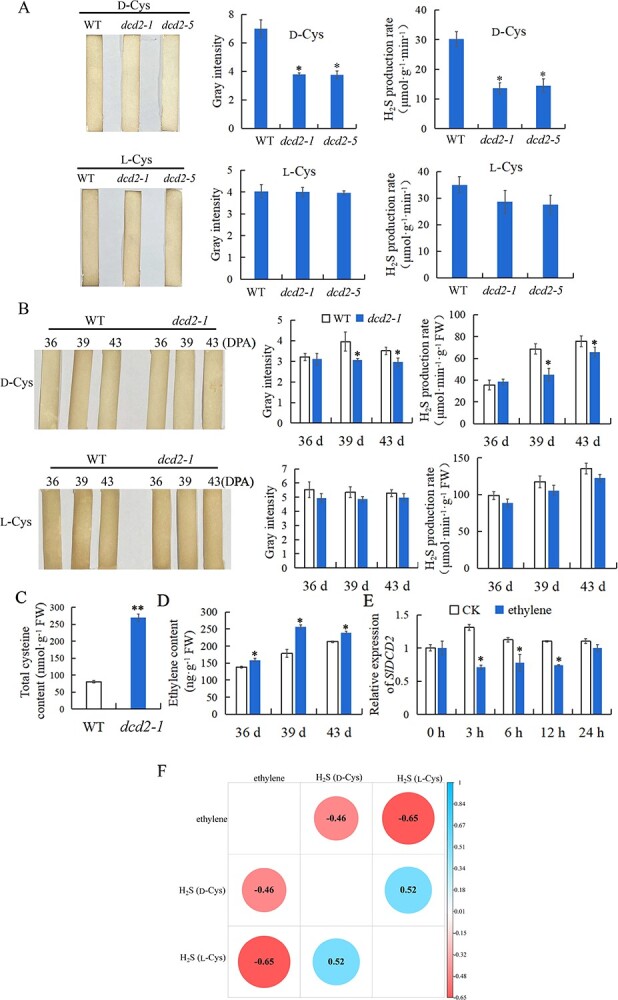
Mutation of *SlDCD2* caused a decrease in the level of H_2_S and increases in the levels of cysteine and ethylene. H_2_S produced by soluble proteins in the mature leaves (**A**) and fruits (**B**) of WT and *dcd2–1/5* lines with D-cysteine or L-cysteine as the substrate was detected using zinc acetate test strips and the methylene blue method. The gray intensity of each test strip in (**A**) and (**B**) was analysed using ImageJ. **C** Determination of the total cysteine content in mature leaves of the WT and *dcd2–1* lines. **D** Changes in the ethylene content in WT and *dcd2–1* fruits at 36, 39, and 43 DPA. **E** Effect of ethylene treatment on the transcript level of *SlDCD2* in WT fruits at 0 h, 3 h, 6 h, 12 h, and 24 h. **F** Correlation analysis of H_2_S produced using L-cysteine and D-cysteine as the substrate with the ethylene content in WT and *dcd2–1* fruits at 36, 39, and 43 DPA. The data are expressed as the means ± SDs of three replicates. ^**^P < 0.01; ^*^P < 0.05.

To confirm the effect of *SlDCD2* mutation on endogenous H_2_S production, we first measured the endogenous H_2_S content in *dcd2-1/5* mutant leaves using lead acetate H_2_S detection strips. The results presented in Fig. 3A show that *dcd2-1/5* mutant leaves produced less H_2_S with D-Cys as the substrate, and their gray intensity in the strips was significantly lower than that found for the WT leaves. We also verified the difference in H_2_S production with L-Cys and D-Cys as substrates using the methylene blue method. The rate of H_2_S production in WT leaves was approximately 1.5 times higher than that in *dcd2* mutant leaves when D-Cys was used as the substrate. However, the difference between *dcd2* mutants and WT was not significant when L-Cys was used as the substrate, and that between *dcd2-1/5* mutant and WT leaves was not significant when L-Cys was used as the substrate (Fig. 3A), suggesting that D-cysteine rather than L-cysteine is the only substrate for SlDCD2. We then measured the endogenous H_2_S content in WT and *dcd2-1* fruits at 36, 39, and 43 DPA using lead acetate H_2_S detection strips. The results showed that *dcd2-1* fruits at 39 and 43 DPA produced less H_2_S with D-Cys as the substrate, and the gray intensity of the strips was significantly lower than that found for the WT (Fig. 3B). In addition, we determined the H_2_S production rate in the fruits using the methylene blue method. The results show that the production rate of H_2_S in the WT fruits at 39 and 43 DPA was approximately 1.3 and 1.2 times higher than that in the *dcd2-1*-mutant fruits at 39 and 43 DPA, respectively (Fig. 3B). When L-Cys was used as the substrate, the difference in the H_2_S content and production rate between WT and *dcd2-1* fruits was not significant, suggesting that the mutation of *SlDCD2* reduces H_2_S production during tomato fruit ripening and accelerates fruit ripening (Fig. 3B).

In addition, due to the ability of SlDCD2 to decompose D-cysteine, we propose that *dcd2-1/5* mutations may cause the accumulation of total cysteine. It is difficult to discriminate L-cysteine from D-cysteine; therefore, we determined the content of total cysteine in tomato leaves. Consistently, Fig. 3C shows that the *dcd2-1* mutation resulted in a significantly higher level of total cysteine than that in WT, implying that the mutation damaged the enzymatic function of SlDCD2 as a DCD.

Previous studies have indicated that H_2_S significantly alleviates fruit ripening and senescence by attenuating the effect of ethylene. The antagonistic effect of H_2_S and ethylene may play an important role in fruit senescence. To test this hypothesis, we measured the ethylene content of WT and *dcd2-1* mutant fruits during ripening and found that the ethylene content in *dcd2-1* fruit at 36, 39, and 43 DPA was significantly higher than that in WT (Fig. 3D). In particular, there was a higher increase in ethylene content in *dcd2-1* fruits at 39 DPA than in WT fruits. Correlation analysis indicated a negative correlation between H_2_S produced by either L-cysteine or D-cysteine as a substrate and ethylene content. Specifically, the correlation between ethylene and H_2_S produced by L-cysteine was −0.65, and the correlation between ethylene and H_2_S produced by D-cysteine was −0.46 (Fig. 3F). Therefore, the mutation of *SlDCD2* affects ethylene biosynthesis, which accelerates fruit ripening.

Subsequently, we treated WT fruits at 36 DPA with 1 g/L ethephon aqueous solution fumigation, and the expression of *SlDCD2* under ethylene treatment was studied by RT–qPCR. The results indicated that ethylene treatment caused decreased expression of *SlDCD2* at 3 h, 6 h, and 12 h compared with the control (Fig. 3E). At 24 h, *SlDCD2* expression in fruits of both groups gradually returned to the pretreatment level (Fig. 3E). Therefore, we found that ethylene decreased the expression of *SlDCD2*.

### Effect of *SlDCD2* mutation on the chlorophyll and carotenoid contents and the expression of ripening-related genes during the fruit ripening process

**Figure 4:**
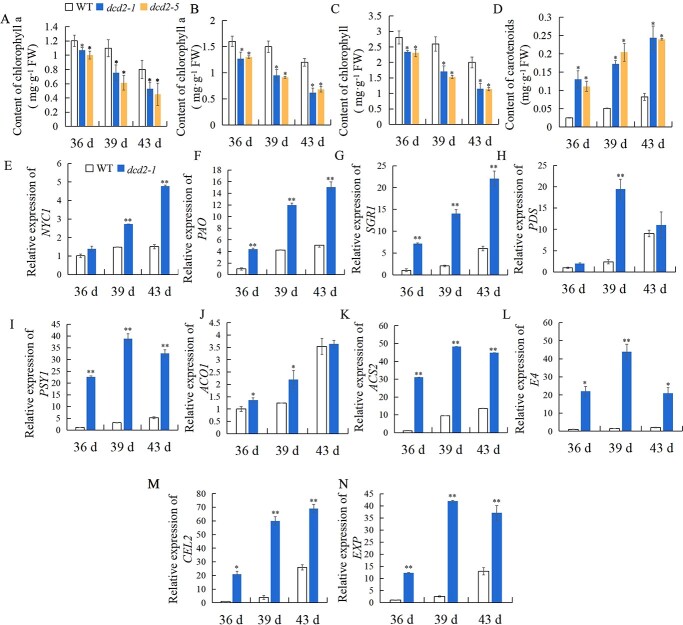
Changes in pigment contents and the expression of ripening-associated genes during tomato fruit ripening. Changes in the contents of (**A**) chlorophyll a, (**B**) chlorophyll b, (**C**) total chlorophyll, and (**D**) carotenoids in WT, *dcd2–1*, and *dcd2–5* tomato fruits at 36, 39, and 43 DPA. Gene expression of *NYC1* (**E**), *PAO* (**F**), *SGR1* (**G**), *PDS* (**H**), *PSY1* (**I**), *ACO1* (**J**), *ACS2* (**K**), *E4* (**L**), *CEL2* (**M**), and *EXP* (**N**) in WT and *dcd2–1* tomato fruits at 36, 39, and 43 DPA. The data are expressed as the means of three biological replicates ± SDs. ^**^P < 0.01; ^*^P < 0.05.

In the *dcd2* mutant, the mutation of *SlDCD2* accelerated fruit ripening; therefore, we determined the chlorophyll and carotenoid contents in the *dcd2-1* and *dcd2-5* fruits at 36, 39, and 43 DPA. Fig. 4A–C show that the contents of chlorophyll a, chlorophyll b, and total chlorophyll decreased gradually from 36 to 43 DPA in the WT, *dcd2-1*, and *dcd2-5* fruits, whereas the chlorophyll content was significantly lower in the *dcd2* mutants than in the WT. Fig. 4D shows an increasing carotenoids content during fruit ripening in the WT and the *dcd2-1* and *dcd2-5* mutants, and the carotenoids content in the *dcd2-1* fruits was approximately 3 and 3.4 times that in the WT fruits at 39 and 43 DPA, respectively. Overall, the two mutant lines *dcd2-1* and *dcd2-5* showed similar accelerated fruit ripening, and the results from the analysis of the metabolism of chlorophyll and carotenoids further imply a negative role of *SlDCD2* in fruit ripening.

To further analyse the role of *SlDCD2* in delaying fruit ripening, the expression patterns of ripening-associated genes in *dcd2-1* and WT fruits were analysed by RT–qPCR. The expression of chlorophyll degradation-related genes including *NYC1* (Fig. 4E), *PAO* (Fig. 4F), and *SGR1* (Fig. 4G) in *dcd2-1* fruits showed an increasing pattern from 36 to 43 DPA, and these expression levels were always higher than those in WT fruits. For instance, the expression of *NYC1*, *PAO*, and *SGR1* in *dcd2-1* fruits at 43 DPA was approximately 3–4 times that in WT fruits. The expression levels of *PDS* and *PSY1*, which encode key enzymes for carotenoids biosynthesis, were also analysed. *PDS* (Fig. 4H) and *PSY1* (Fig. 4I) increased gradually in WT fruits during ripening, but their expression increased significantly in *dcd2-1* fruits.

The ripening of tomato fruit, as a respiratory climacteric fruit, relies heavily on ethylene synthesis and response pathways. Fig. 4J shows that the expression of *ACO1* increased in both the WT and *dcd2-1* fruits during fruit ripening, whereas *dcd2* mutation resulted in significantly higher expression of *ACO1* at 36 and 39 DPA compared with that in the WT fruits. The expression of *ACS2* (Fig. 4K) and *E4* (Fig. 4L) increased during fruit ripening in the WT fruits, and significantly higher levels of *ACS2* and *E4* were observed in *dcd2-1* fruits. The expression of *CEL2* (Fig. 4M) and *EXP* (Fig. 4N), which are needed for cell wall metabolism, was also analysed. As shown in Fig. 6I and J, the expression of *CEL2* and *EXP* in *dcd2-1* fruits was approximately 12 and 10 times that in the WT fruits, respectively, at 39 DPA. Based on the above-described results, the mutation of *SlDCD2* increased the expression of ripening-associated genes during the tomato fruit ripening process, resulting in accelerated fruit ripening.

### Effects of *SlDCD2* mutation on ROS homeostasis

**Figure 5:**
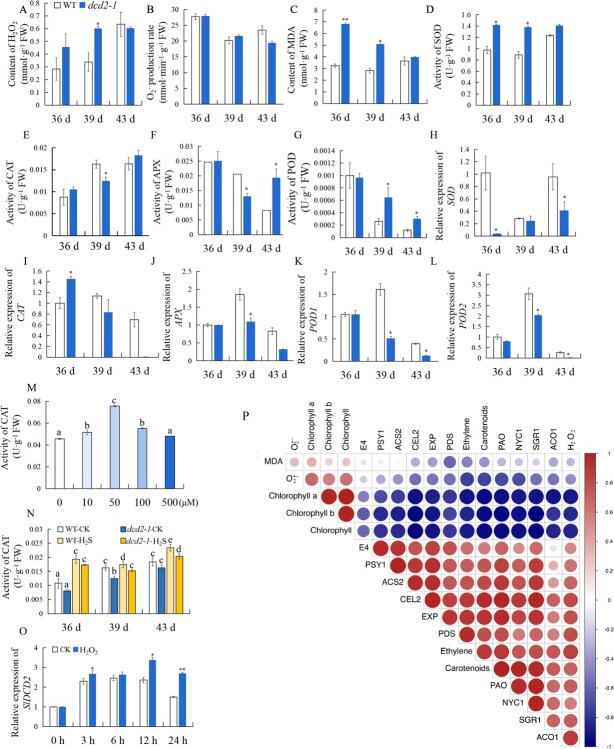
Effect of *SlDCD2* mutation on ROS homeostasis during tomato fruit ripening. **A**−**C** Content of H_2_O_2_ (**A**), production of O_2_^•−^ (**B**) and content of MDA (**C**) in WT and *dcd2–1* tomato fruits at 36, 39, and 43 DPA. **D**−**G** Activities of SOD (**D**), CAT (**E**), APX (**F**), and POD (**G**) in WT and *dcd2–1* tomato fruits at 36, 39, and 43 DPA. **H**−**L** Gene expression of the antioxidant enzyme-related genes *SOD* (**H**), *CAT* (**I**), *APX* (**J**), *POD1* (**K**), and *POD2* (**L**) in WT and *dcd2–1* fruits at 36, 39, and 43 DPA. **M** Effect of 0, 10, 50, 100, and 500 μM NaHS solution treatment on CAT activity in the WT leaves. **N** Effect of 0, 10, 50, 100, and 500 μM NaHS solution treatment on CAT activity in the WT and *dcd2–1* tomato fruits at 36, 39, and 43 DPA. **O** Effect of 5 mmol/L H_2_O_2_ treatment on the expression level of *SlDCD2* in WT fruits at 0 h, 3 h, 6 h, 12 h, and 24 h. (**P**) Correlation analysis among various parameters, including total chlorophyll, chlorophyll a, chlorophyll b, carotenoid contents, H_2_O_2_ content, production of O_2_^•−^, MDA content, ethylene content and gene expression of *NYC1*, *PAO*, *SGR1*, *PDS*, *PSY1*, *ACO1*, *ACS2*, *E4*, *CEL2*, and *EXP* in WT and *dcd2–1* tomato fruits at 36, 39, and 43 DPA. The data are presented as the means of three biological replicates ± SDs. ^**^P < 0.01; ^*^P < 0.05. Significant differences at the level of *P* < 0.05 are indicated with letters.

ROS in plants can be generated as byproducts during normal metabolic processes or as responses to stress conditions. ROS are also thought to be important impact factors of fruit ripening. The exogenous application of H_2_S could decrease ROS overaccumulation in multiple plant species; thus, we propose that *SlDCD2* may delay fruit ripening by modulating ROS homeostasis. As shown in Fig. 5A, the H_2_O_2_ level in both WT and *dcd2-1* fruits increased significantly during fruit ripening, whereas the level in *dcd2-1* fruits was significantly higher than that in WT fruits at 39 DPA. The production of O_2_^•−^ was not significantly different between WT and *dcd2-1* fruits (Fig. 5B). As shown in Fig. 5C, MDA, a product of lipid peroxidation, did not show marked changes in WT fruits during ripening but tended to decrease in *dcd2-1* fruits, but the content of MDA in *dcd2-1* fruits was significantly higher than that in WT fruits at 36 and 39 DPA.

The activities of antioxidative enzymes, including superoxide dismutase (SOD), catalase (CAT), ascorbate peroxidase (APX), and peroxidase (POD), were analysed. Fig. 5D shows that SOD activity in *dcd2-1* fruits was always higher than that in WT fruits during fruit ripening. CAT and APX activity could help alleviate oxidative damage by decomposing H_2_O_2_ to below toxic levels. CAT activity was increased in WT fruits at 36 and 39 DPA but decreased in *dcd2-1* fruits at 39 DPA to a level that was about 75% of that in WT fruits (Fig. 5E). A decreasing trend of APX activity was observed in WT fruits during fruit ripening (Fig. 5F). Compared with the results obtained for the WT fruits, *SlDCD2* mutation caused a significantly lower level of APX activity at 39 DPA and a significantly higher level at 43 DPA. POD activity in both WT and *dcd2-1* fruits decreased gradually during fruit ripening, whereas the activity in *dcd2-1* fruits was significantly higher than that in WT fruits (Fig. 5G). Moreover, the expression of genes encoding antioxidative enzymes, including *SOD*, *CAT*, *APX*, *POD1*, and *POD2* was analysed. As shown in Fig. 5H, SOD expression was lower in *dcd2-1* fruits at 36 and 43 DPA. As shown in Fig. 5I, a decreasing trend of CAT expression was observed in WT and *dcd2-1* fruits, and *SlDCD2* mutation caused significantly higher CAT expression at 36 DPA and significantly lower CAT expression at 43 DPA compared with the results observed in the WT fruits. As shown in Fig. 5J, K, and L, the expression patterns of *APX*, *POD1*, and *POD2* were similar during fruit ripening. Their expression increased at 39 DPA and then decreased at 43 DPA, and the expression levels of *APX*, *POD1*, and *POD2* were significantly lower in *dcd2-1* fruits than in WT fruits.

Overall, *SlDCD2* mutation induced excessive accumulation of H_2_O_2_ and MDA, suggesting that the lower level of H_2_S in *dcd2-1* fruits may lead to an imbalance in ROS metabolism and that excessive ROS may accelerate fruit ripening. As the primary enzyme for the decomposition of H_2_O_2_, CAT undergoes persulfidation in the leaves of Arabidopsis [37, 38]. In the current study, decreased CAT activity was observed in *dcd2-1* fruits; thus, we propose that an appropriate endogenous level of H_2_S may be needed for altering the activity of CAT. CAT extracted from tomato leaf tissue was treated with different levels of H_2_S, and the results in Fig. 5M indicate that 50 μM NaHS (H_2_S donor) could activate CAT. WT and *dcd2-1* mutant fruits were also treated with 50 μM NaHS, and the results showed that CAT activity was enhanced (Fig. 5N). Subsequently, tomato fruits at 36 DPA were treated with 5 mmol/L H_2_O_2_ to evaluate the potential interaction between H_2_O_2_ and *SlDCD2*. The expression of *SlDCD2* in H_2_O_2_-treated fruits after 3 h and 6 h of treatment was not significantly different from that in the control fruits, but at 12 and 24 h, the expression in the H_2_O_2_-treated fruits was significantly higher than that in the control fruits (Fig. 5O). Therefore, exogenous H_2_O_2_ application to tomato fruits stimulated the expression of *SlDCD2*, which may lead to an increase in H_2_S production in the fruits and thereby alleviate H_2_O_2_-induced oxidative stress.

To reveal the potential associations among the parameters, then correlations among the chlorophyll a, chlorophyll b, total chlorophyll, carotenoids content, and H_2_O_2_ contents, O_2_^•−^ production rate, MDA content, and expression of ripening-related genes were analysed. As shown in Fig. 5P, the chlorophyll content was negatively correlated with the expression of ripening and senescence-related genes, and the carotenoids content was positively related to the expression of these genes. Moreover, the chlorophyll content was negatively correlated with the H_2_O_2_ content, whereas no obvious correlation was found for the chlorophyll content with the O_2_^•−^ production rate and MDA content. In addition, H_2_O_2_ is positively correlated with the ethylene content, the carotenoids content and the expression of all ripening-related genes, including *NYC1*, *PAO*, *SGR1*, *PDS*, *PSY1*, *ACO1*, *ACS2*, *E4*, *CEL2*, and *EXP.* More importantly, H_2_O_2_ may be the key ROS that promotes fruit ripening because a strong positive correlation was found for H_2_O_2_ with the carotenoids content, ethylene content and ripening-related gene expression. However, the production rate of O_2_^•−^, which is also a form of ROS, and the MDA content were not significantly related to fruit ripening.

### Effect of *SlDCD2* mutation on ROS homeostasis in tomato leaves

**Figure 6:**
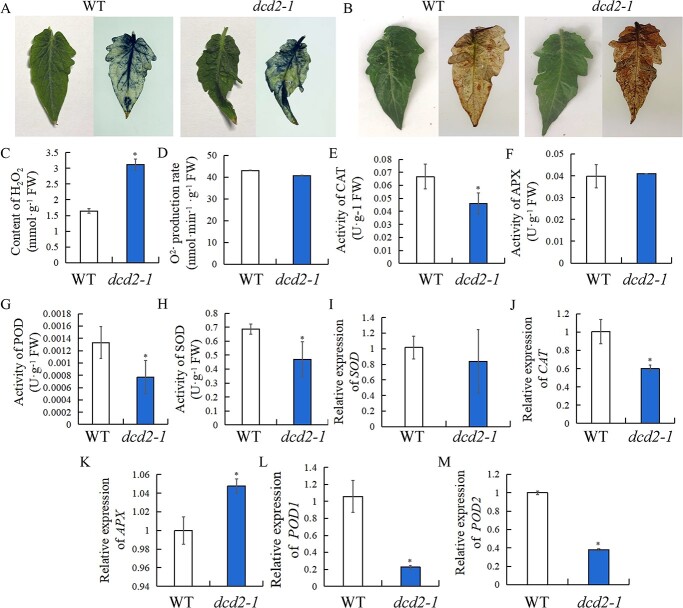
Effect of *SlDCD2* mutation on ROS homeostasis in tomato leaves. **A** WT and *dcd2–1* tomato leaves stained with NBT. **B** WT and *dcd2–1* tomato leaves stained with DAB. **C**−**D** Content of H_2_O_2_ and production of O_2_^•−^ in WT and *dcd2–1* tomato leaves. **E**−**H** Activities of CAT (**E**), APX (**F**), POD (**G**), and SOD (**H**) in WT and *dcd2–1* tomato leaves. **I**−**M** Gene expression levels of the antioxidant enzyme-related genes *SOD* (**I**), *CAT* (**J**), *APX* (**K**), *POD1* (**L**), and *POD2* (**M**) in WT and *dcd2–1* tomato leaves. The data are presented as the means of three biological replicates ± SDs. ^**^P < 0.01; ^*^P < 0.05.

To investigate the universal role of H_2_S generated by DCD in modulating ROS homeostasis and the MDA content, the activities of antioxidative enzymes and their gene expression levels in WT and *dcd2-1* tomato leaves were determined. As shown in Fig. 6A, *SlDCD2* mutation caused accelerated leaf yellowing after 60 days of growth compared with the results found for WT leaves, suggesting that *SlDCD2* mutation caused earlier leaf senescence. Nitrotetrazolium blue chloride (NBT) staining and diaminobenzidine (DAB) staining were used to visualize the distribution of O_2_^•−^ and H_2_O_2_ in leaves, respectively. The accumulation of O_2_^•−^ in WT and *dcd2-1* leaves did not differ significantly based on NBT staining (Fig. 6A), but a higher level of H_2_O_2_ was found in *dcd2* mutant leaves as revealed by DAB staining (Fig. 6B). The results indicated that mutation of *SlDCD2* resulted in higher accumulation of H_2_O_2_ in leaves. The results from the analysis of the H_2_O_2_ content shown in Fig. 6C suggested that *SlDCD2* mutation resulted in increased H_2_O_2_ in comparison to the levels found in WT plants, whereas the O_2_^•−^ production rate did not significantly differ between WT and *dcd2-1* tomato leaves (Fig. 6D). Additionally, *SlDCD2* mutation decreased the activities of CAT (Fig. 6E), POD (Fig. 6G), and SOD (Fig. 6H) but did not significantly change the activity of APX (Fig. 6F). Furthermore, *SlDCD2* mutation caused significantly lower gene expression of *CAT* (Fig. 6J), *POD1* (Fig. 6L), and *POD2* (Fig. 6M) and higher expression of *APX* (Fig. 6K) and did not significantly change the expression of *SOD* (Fig. 6I) compared with the levels found in the WT plants. Overall, the results indicate that *SlDCD2* mutation also accelerated leaf senescence, which may be due to disturbed ROS homeostasis.

## Discussion

In our previous study, the *SlLCD1* gene-edited mutant displayed accelerated fruit ripening and earlier leaf senescence, suggesting that *SlLCD1* is a negative impact factor of fruit ripening and leaf senescence [26]. However, the role of DCD in tomato fruit ripening and leaf senescence remains unclear. In the present work, the proteins in *S. lycopersicum*, *A. thaliana*, *V. vinifera*, and *G. max* that are homologous to AtDCD1 (AT1G48420) were obtained. SlDCD1/2 show high homology with homologs in grapevine, and SlDCDs are evolutionarily closer to VvDCDs. Previous studies have shown that the application of exogenous H_2_S effectively alleviates postharvest senescence in grape [39, 40], but whether DCD influences grape ripening and senescence is unclear.

Because *SlDCD2* expression shows an increasing trend during fruit ripening, we propose that *SlDCD2* may be involved in fruit ripening. First, the expression of *SlDCD2* was transiently silenced by VIGS, and accelerated fruit ripening was observed in TRV2-*SlDCD2* fruits compared with fruits carrying the empty vector. The expression of *SlDCD2* in *SlDCD2*-slicenced fruits was approximately half that found in the control fruits, suggesting the efficiency of the gene silencing. Additionally, the chlorophyll content in the *SlDCD2*-silenced fruits degraded more rapidly than that in the control fruits, and greater carotenoids accumulation was detected in *SlDCD2*-silenced fruits. In addition, *SlDCD2* silencing increased the expression of the chlorophyll degradation genes *NYC1*, *PAO*, and *SGR1*, the carotenoids synthesis genes *PSY1* and *PDS*, the ethylene synthesis genes *ACO1*, *ACS2*, and *E4*, and the cell wall metabolism gene *CEL2*, suggesting the potential negative role of *SlDCD2* in fruit ripening.

The role of *SlDCD2* was confirmed by stable *SlDCD2* gene editing of tomato lines using CRISPR/Cas9 technology. Both *dcd2-1* and *dcd2-5* showed decreased H_2_S release, accelerated fruit ripening and an increase in the content of cysteine. D-cysteine and L-cysteine were supplied as substrates for the *dcd2-1* and *dcd2-5* mutants, and the mutants only showed decreased H_2_S production when the substrate was D-cysteine. In addition, the substrate selectivity was confirmed by purified SlDCD2 protein, which only used D-cysteine as the substrate. However, AtDCD2 was found to catalyze the production of H_2_S from both D-cysteine and L-cysteine [19], suggesting that the function of DCD2 is not highly conserved in Arabidopsis and tomato. The levels of chlorophyll and carotenoids in the *dcd2* mutant fruits were analysed, and the results indicated that *SlDCD2* mutation caused accelerated chlorophyll degradation and premature carotenoids biosynthesis.

Previous studies indicate that H_2_S delays fruit ripening by antagonizing the effect of ethylene. The results show that H_2_S prolongs the postharvest ripening and senescence of banana during storage by antagonizing the effect of ethylene [11]. In the present study, the ethylene content in *dcd2-1* fruits was significantly higher than that in WT fruits. The correlation analysis indicated a negative correlation between H_2_S produced with either L-cysteine or D-cysteine as the substrate and the ethylene content. In addition, exogenous ethylene treatment decreased the expression of *SlDCD2* compared with that obtained with the control treatment. Therefore, mutation of *SlDCD2* may induce enhanced ethylene biosynthesis and thereby accelerate fruit ripening, which further suggests the antagonizing effect between H_2_S and ethylene.

The effect of reduced H_2_S on the expression of ripening-associated genes was also evaluated in the *dcd2* mutant. The expression of *NYC1*, *SGR1*, and *PAO* was highly upregulated during *dcd2* fruit ripening. In addition, the expression of the carotenoids biosynthesis genes *PSY1* and *PDS* was upregulated in *dcd2* mutant fruits. Tomato fruit ripening is also accompanied by increases in ethylene synthesis and cell wall degradation. The present study indicates that *ACO1*, *ACS2*, and *E4* were all expressed at obviously higher levels in *dcd2* than that in WT tomato fruits. Our data also show that the *dcd2* mutant showed increased expression of *CEL2* and *EXP.*

Exogenous H_2_S was found to delay the ripening and senescence of multiple plants by reducing excessive ROS [5–7]. The production of ROS during fruit ripening and senescence is natural and unavoidable due to imbalanced metabolism. However, how endogenous H_2_S affects ROS homeostasis remains unclear. Therefore, we determined the ROS content during fruit ripening. The data indicate that H_2_O_2_ was significantly increased in both WT and *dcd2-1* fruits during fruit ripening and that the content in *dcd2-1* fruits was clearly higher than that in WT fruits. The production of O_2_^•−^ did not significantly differ between WT and *dcd2-1* plants. The *dcd2-1* mutants also showed increased MDA contents compared with the WT at 36 and 39 DPA. In a previous study, Pilati *et al.* [33] observed a rapid accumulation of H_2_O_2_ during grape ripening, which may be associated with grape softening and ripening. In addition, the H_2_O_2_ levels linearly increased as the nectarine fruit developed and ripened on trees [32]. The promoting effect of H_2_O_2_ on fruit ripening has also been observed in pears [41]. In the present study, H_2_O_2_ was increased at 39 DPA in *dcd2-1* fruits, which had just completed their transition from the breaker to the red stage. Consistently, previous studies observed elevations in the H_2_O_2_ levels in tomatoes, which also exhibited changes in their skin color [42]. Therefore, fruit ripening appears to be positively associated with H_2_O_2_. The results indicate that H_2_O_2_ is the ROS signal that participates in the tomato fruit ripening process. Additionally, the enzymatic activities of SOD, CAT, APX, and POD were analysed, and the results showed that SOD activity in *dcd2-1* fruits was always higher than that in WT fruits during fruit ripening. CAT and APX activity could help alleviate oxidative damage by reducing the H_2_O_2_ levels to below toxic levels, and compared with that in the WT plants, *dcd2* mutation resulted in significant decreases in CAT and APX activities at 36 DPA. POD activity in both WT and *dcd2-1* fruits decreased gradually during fruit ripening, whereas the activity in *dcd2-1* fruits was significantly higher than that in WT fruits. SOD catalyzes the dismutation of O_2_^•−^ to molecular oxygen and H_2_O_2_ [43], and CAT decomposes H_2_O_2_ [44]. The levels of O_2_^•−^ and H_2_O_2_ are controlled by the activity of different antioxidative enzymes. As revealed in the current study, *dcd2* mutation caused higher SOD activity and lower CAT activity, which may act synergistically to produce excessive H_2_O_2_. In addition, the H_2_O_2_ level increased significantly during fruit ripening, which also supports the role of H_2_O_2_ in fruit ripening. Overall, *SlDCD2* mutation caused H_2_O_2_ and MDA accumulation, suggesting that the lower level of H_2_S in *dcd2-1* may lead to an imbalance in ROS metabolism and that excessive H_2_O_2_ may accelerate fruit ripening. As CAT undergoes persulfidation in the leaves of Arabidopsis [37, 38], we found decreased CAT activity in *dcd2-1* fruits; thus, we propose that an appropriate endogenous level of H_2_S may provide a microenvironment favorable for the activity of CAT. The results depicted in Fig. 5M and N show that 50 μM NaHS could enhance the activity of CAT in tomato tissues, suggesting that endogenous H_2_S enhances CAT activity. However, whether the increased CAT activity is caused by protein persulfidation still needs further research.

To reveal the potential association among the parameters, the correlations among the chlorophyll a, chlorophyll b, total chlorophyll, and carotenoid contents, H_2_O_2_ content, O_2_^•−^ production rate, MDA content and gene expression of *NYC1*, *PAO*, *SGR1*, *PDS*, *PSY1*, *ACO1*, *ACS2, E4, CEL2*, and *EXP* were analyzed. The results indicate that the chlorophyll content was negatively correlated with the expression of ripening- and senescence-related genes, whereas the carotenoids content was positively related to the expression of these genes. Moreover, the chlorophyll content was negatively correlated with the H_2_O_2_ content, but no obvious correlation was found between the O_2_^•−^ production rate and the MDA content. In addition, H_2_O_2_ was positively correlated with the carotenoids content and the expression of all ripening-related genes*.* Therefore, H_2_O_2_ is the key ROS that promotes fruit ripening because a strong positive correlation was found between H_2_O_2_ and the carotenoids content and ripening-related gene expression. However, the O_2_^•−^ production rate and MDA content were not significantly related to fruit ripening. Therefore, we provide strong evidence showing that H_2_O_2_ is the ROS that modulates fruit ripening and that H_2_S reduction is associated with excessive H_2_O_2_ accumulation. The interaction between H_2_S and H_2_O_2_ is complex. H_2_S was found to facilitate the generation of H_2_O_2_ by increasing NADPH oxidase at the transcriptional and enzymatic levels [36, 45, 46]. However, the function of H_2_S in fruit ripening involves the removal of excessive H_2_O_2_. The role of H_2_S in modulating ROS homeostasis was also studied in *dcd2* leaves, and the data indicate that the *dcd2* mutant exhibited earlier leaf senescence. In addition, *dcd2* mutation caused a higher level of H_2_O_2_, whereas the production of O_2_^•−^ did not significantly differ between WT and *dcd2* leaves.

In summary, we found that *SlDCD2* is involved in H_2_S generation and that the reduction in endogenous H_2_S results in accelerated fruit ripening and earlier leaf senescence. Additionally, the decreased H_2_S level in the *dcd2* mutant caused excessive H_2_O_2_ accumulation and increased ethylene release, suggesting the role of H_2_S and *SlDCD2* in regulating ROS homeostasis and ethylene biosynthesis. In addition, we provide strong evidence showing that H_2_O_2_ is the key form of ROS that participates in fruit ripening.

## Materials and methods

### Phylogenetic analysis

Putative DCD proteins in *S. lycopersicum*, *A. thaliana*, *V. vinifera*, and *G. max* were obtained with the BLASTP tool in the Phytozome v13 (https://phytozome.jgi.doe.gov/pz/portal.html#) database using AtDCD1 (AT1G48420) as the query. The amino acid sequences of SlDCD1 and SlDCD2 from *S. lycopersicum*; AtDCD1 and AtDCD2 from *A. thaliana*; VvDCD1, VvDCD2, and VvDCD3 from *V. vinifera*; and GmDCD1 and GmDCD2 from *G. max* were selected to construct a phylogenetic tree using the neighbor-joining method according to the parameters previously reported by Saitou *et al*. [47].

### Expression, purification, and enzymatic activity assay of the recombinant protein SlDCD2 in *E. coli*

The 1329-bp coding sequence of *SlDCD2* (accession number: Solyc01g008900) was amplified using the primers listed in Table S1 (see online supplementary material). The coding sequence of *SlDCD2* was then ligated into the pCOLD vector, and the confirmed pCOLD-*SlDCD2* plasmid was transformed into DE3 cells. Subsequently, the expression, collection, and purification of the SlDCD2 protein were performed using the methods described by Cheng *et al*. [48]. The expression and purification of the SlDCD2 protein were confirmed by SDS–PAGE and staining with Coomassie Blue. The detection of the SlDCD2 protein was verified by Western blot using anti-His antibody.

L/D-Cysteine was used as the substrate as previously described [49], and the enzymatic activity of DCD was determined at 670 nm. Standard curves were prepared using solutions of NaHS at different concentrations.

### Transient silencing of *SlDCD2* in tomato fruit

The fragment (with a length of 400 bp corresponding to nt 1–400) encoding *SlDCD2* was amplified and inserted into the pTRV2 plasmid to yield recombinant pTRV2-*SlDCD*2. The fragment of the *SlDCD2* gene was amplified with specific primers, which are listed in Table S1 (see online supplementary material). As described previously by Fantini *et al*. [50], Agrobacterium containing the pTRV1 vector and the corresponding pTRV2 vector was inoculated into pedicels of tomato plants after mixing at a ratio of 1:1, and infected tomato petioles infiltrated with the empty pTRV2 vector were used as controls. Tomato (*S. lycopersicum*, Micro Tom) plants were first incubated in the dark at 16°C for 24 h and then incubated under the following conditions: 16-h day/8-h night cycle, 25 ± 2°C/20 ± 2°C day/night temperature, 65% relative humidity, and 250 μmol m^−2^ s^−1^ light intensity. The appearance of the tomato fruit color was assayed using a color difference meter (model WSC-100; Konica Minolta, Tokyo, Japan).

### Generation and genotyping of the *dcd2* mutant by CRISPR/Cas9

CRISPR/Cas9 mutagenesis of *SlDCD2* in tomato was performed as previously described [51–53]. The primers for sgRNA are listed in Table S1 (see online supplementary material). For confirmation of the *dcd2* mutant, we amplified a fragment of the sgRNA target sequence using genomic DNA from the *dcd2* mutant. The amplification product was used for DNA sequencing, and the genotyping of tomato plants was analysed on the website DSDecodeM (http://skl.scau.edu.cn/dsdecode/) [54].

mRNA from WT and *dcd2* mutants were extracted and reverse transcribed into cDNA, and the cDNA was used as the template for amplification of the CDS of *SlDCD2*. The amplification products were subsequently sequenced. The sequencing peak maps were analysed using Chromas software.

### Treatment of tomato fruit with pyruvate, NH_4_Cl, or NaHS

WT tomato fruits at the white mature stage were soaked with H_2_O, 50 μM NaHS solution (H_2_S treatment group), 50 μM pyruvate solution, or 50 μM ammonium chloride solution for 8 h. Subsequently, the fruits were dried and placed on wet sterile filter papers in Petri dishes. The Petri dishes, which contained 10 fruits, were stored at 23°C for 4 days and photographed.

### Treatment of tomato fruit using H_2_O_2_ or ethylene

According to previous studies [11, 55], tomato fruits at 36 DPA were fumigated with 1 g/L ethephon aqueous solution or sprayed with 5 mmol/L H_2_O_2_. Fruit samples collected after 0 h, 3 h, 6 h, 12 h, and 24 h of treatment were used for mRNA extraction and qPCR.

### RNA extraction and RT–qPCR

Reactions were conducted using previously reported methods [9]. Tomato **SlT*ubulin* was used as an internal reference. The primers used for RT–qPCR analysis are listed in Table S1 (see online supplementary material).

### Determination of the levels of chlorophyll, carotenoids, and cysteine in tomato fruit

A tomato fruit sample (0.5 g) without seeds was extracted in ethanol, and quantitative determination of the chlorophyll and carotenoid contents was conducted. The chlorophyll and carotenoids levels were measured and calculated based on the equations described by Wellburn [56].

A cysteine assay kit (Solarbio, Beijing, China) was used to determine the content of cysteine. Samples (0.2 g) of WT and *dcd2* tomato leaves were used for cysteine determination according to the manufacturer’s instructions.

### Determination of the amount of H_2_S release in tomato leaves and gray intensity analysis

As mentioned previously [9], the release of H_2_S in 1.0 g of leaf or fruit samples was determined using zinc acetate test strips (Sigma, Darmstadt, Germany). The amount of H_2_S release is related to the color of the zinc acetate test strips. A gray intensity analysis of the zinc acetate test strips was performed using ImageJ software.

The release of H_2_S in 1.0 g of leaf or fruit samples was also determined using the methylene blue spectrophotometric method [57, 58]. Leaf or fruit samples were ground to a powder in liquid nitrogen, homogenized in 5 mL of buffer (containing 100 mM potassium phosphate buffer pH 7.4, 10 mM Cys, and 2 mM pyridoxal 5′-phosphate) and then centrifuged to obtain the supernatant. After centrifugation, the released H_2_S was adsorbed with zinc acetate and further reacted with N,N-dimethyl-phenylenediamine (DPD) with FeCl_3_ to form methylene blue, which was detected colorimetrically at 670 nm.

### Determination of the ethylene content

The ethylene content was determined as described by Xie *et al*. [59] using the plant ethylene ELISA kit (ColorfulGene, Wuhan, China) and following the manufacturer’s instructions. The content was expressed as ng/g of fresh weight (FW).

### Antioxidant enzyme assay

The crude enzyme solution was prepared according to a previous study [60]. The activities of CAT, APX, SOD, and POD were measured and calculated spectrometrically [61–64]. An absorbance increase of 0.01 OD_470_ nm min^−1^ was considered 1 U of POD activity, a decrease in absorbance of 0.01 at OD_240_ nm min^−1^ was considered 1 U of CAT activity, the amount used to inhibit 50% of the photochemical reduction of NBT was considered 1 U of SOD activity, and a decrease in absorbance of 0.01 at OD_290_ nm min^−1^ was considered 1 U of APX activity. The results are expressed on a FW basis as U·g^−1^.

### MDA content

As mentioned previously [65], 0.5 g of the plant sample was homogenized, incubated, and then centrifuged to collect the supernatant. The absorbance at 450, 532, and 600 nm was measured.

### H_2_O_2_ content

A 0.5-g sample of plant material was homogenized and centrifuged to collect the precipitate. The precipitate was then added to 1.5 mL of 2 M H_2_SO_4_. The absorbance of the mixture was measured at 412 nm, and the content of H_2_O_2_ was calculated [66, 67].

### Production rate of O_2_^•−^

The reaction buffer comprised 50 mM phosphate buffer (pH 7.8) containing 17 mM sulfanilic acid, 1 mM hydroxylamine hydrochloride, 7 mM 1-naphthylamine, and 50-μL sample extracts. The absorbance of the mixture was measured at 530 nm, and the production rate of O_2_^•−^ was calculated using previously described formulas [60].

### Detection of H_2_O_2_ and O_2_^•−^ in tomato leaves

The distribution of O_2_^•−^ was detected as described previously [25]. Briefly, WT and *dcd2* leaves were vacuum infiltrated with 0.1 mg/mL NBT in 25 mM HEPES buffer (pH 7.6) for 1 min in darkness. Chlorophyll was removed using ethanol, and the leaves were photographed.

WT and *dcd2* mature leaves were stained with DAB according to a previously described method [25]. The brown substance formed by the reaction of H_2_O_2_ and DAB reflected the presence and distribution of H_2_O_2_. The leaves were soaked in staining solution (containing 0.05 g of DAB, 25 μL of Tween-20, 2.5 mL of 200 mM Na_2_HPO_4_, and 45 mL of H_2_O, pH 3.0), vacuumed 2–3 times for 1 min, and then rinsed with ethanol for several hours until all the chlorophyll was removed, and the leaves were then photographed.

### Statistical analysis

The data were calculated from three replicates in each experiment, and the experiments were repeated independently three times. Statistical significance was assayed by one-way analysis of variance using IBM SPSS Statistics (SPSS version 20.0; Armonk, NY, USA), and the results are expressed as the means ± SDs. Significant differences were calculated by a *t* test (*P* < 0.01 or *P* < 0.05). The different letters above the columns represent significant differences between two values (*P* < 0.05) at the same time-point.The correlations among the chlorophyll, chlorophyll a, chlorophyll b, and carotenoid contents, H_2_O_2_ content, production of O_2_^•−^, MDA content and gene expression of *NYC1*, *PAO*, *SGR1*, *PDS*, *PSY1*, *ACO1*, *ACS2*, *E4*, *CEL2*, and *EXP* in the WT and *dcd2–1* tomato fruits at 36, 39, and 43 DPA were analysed using the OmicShare platform (https://www.omicshare.com). The correlations among the H_2_S and ethylene content in the WT and *dcd2–1* tomato fruits at 36, 39, and 43 DPA were analysed using the OECloud tools at https://cloud.oebiotech.cn.

## Acknowledgments

This research was supported by the National Natural Science Foundation of China (31970312, 31970200, 32170315, 31901993), the Fundamental Research Funds for the Central Universities (JZ2021HGPA0063), the National Key R&D Program of China (2019YFD1000700, 2019YFD1000701), the National Key R&D Program of China (2019YFD1001300, 2019YFD1001303), and the Natural Science Foundations of Anhui Province (1908085MC72).

## Authors’ contributions

Y.-Q.Z., K.-D.H., and H.Z. conceived and supervised the experiments. Y.-Q.Z., K.-D.H. G.-F.Y., and X.-J.P. performed the research. Y.-Q.Z., K.-D.H., S.-Y.W., and G.-F.Y. analysed the data. Y.-Q.Z., K.-D.H., and H.Z. wrote the paper.

## Data availability

All data supporting the findings of this study are available within the paper and within its supplementary materials published online. The sequence data discussed in this article can be found in the Phytozome v13 database (https://phytozome-next.jgi.doe.gov/).

## Conflict of interest statement

The authors declare that they have no conflict of interest.

## Supplementary data

Supplementary data is available at *Horticulture Research* online.

## Supplementary Material

Web_Material_uhad014
