# Supplementary material for: A D-cysteine desulfhydrase, SlDCD2, participates in tomato fruit ripening by modulating ROS homoeostasis and ethylene biosynthesis
Source: Hortic Res. 2023 Feb 1;10(3):uhad014. doi: 10.1093/hr/uhad014 (PMC10031741; doi:10.1093/hr/uhad014)
Supplement: Web_Material_uhad014 [file web_material_uhad014.zip › supplementary data-dcd2-1.19.docx]

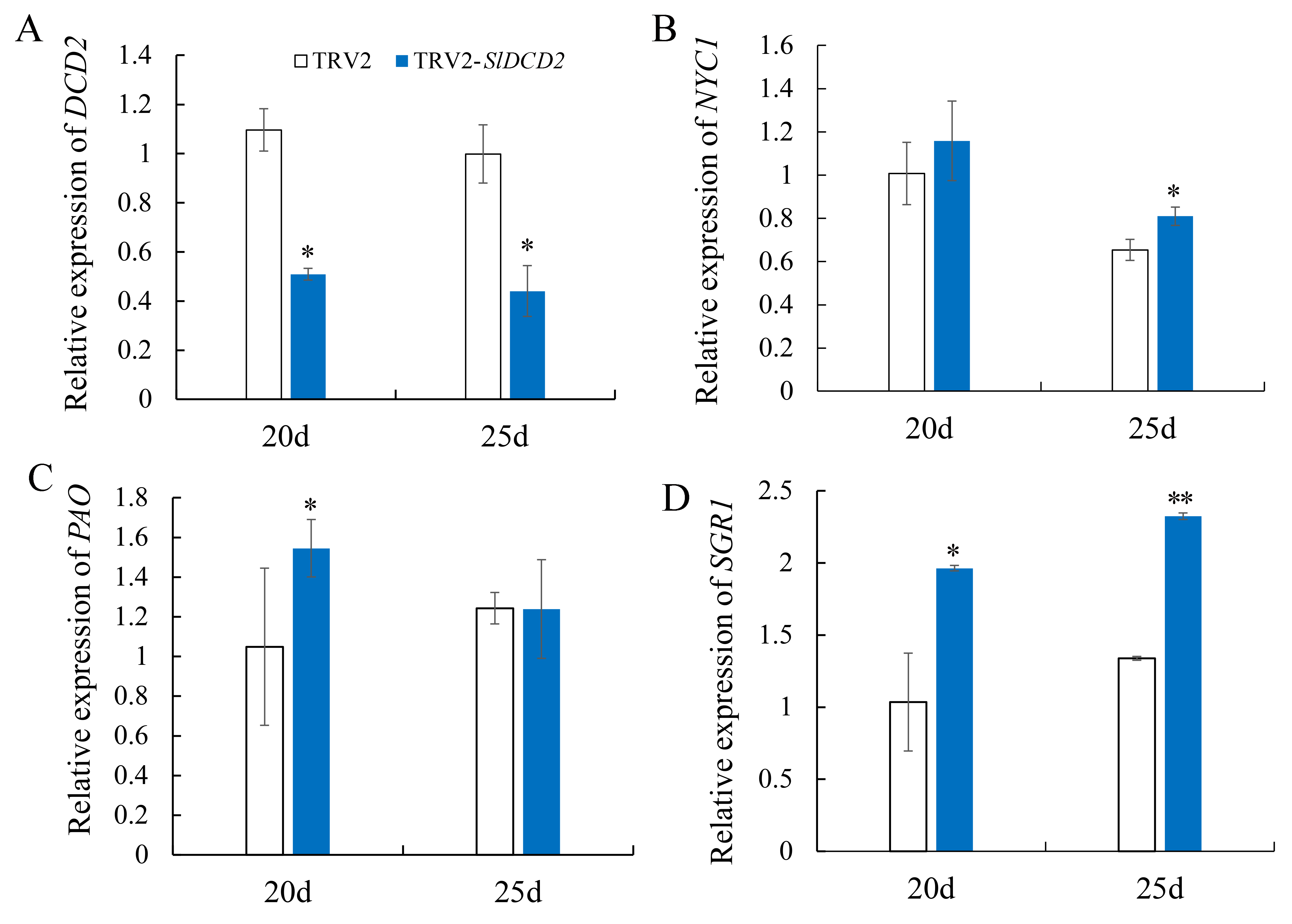


**Figure S1.** The expression of *SlDCD2* (A) and chlorophyll degradation related genes *NYC1* (B), *PAO* (C) and *SGR1* (D) in fruits of wild type (WT) and *SlDCD2*-silenced tomatoes for 20 and 25 days after infection with *Agrobacterium tumefaciens* strain GV3101 containing the recombinant TRV2-*SlDCD2*. The data are expressed as the means of three biological replicates ± SDs. The symbols ** and * indicate *P* < 0.01 and *P* < 0.05, respectively.


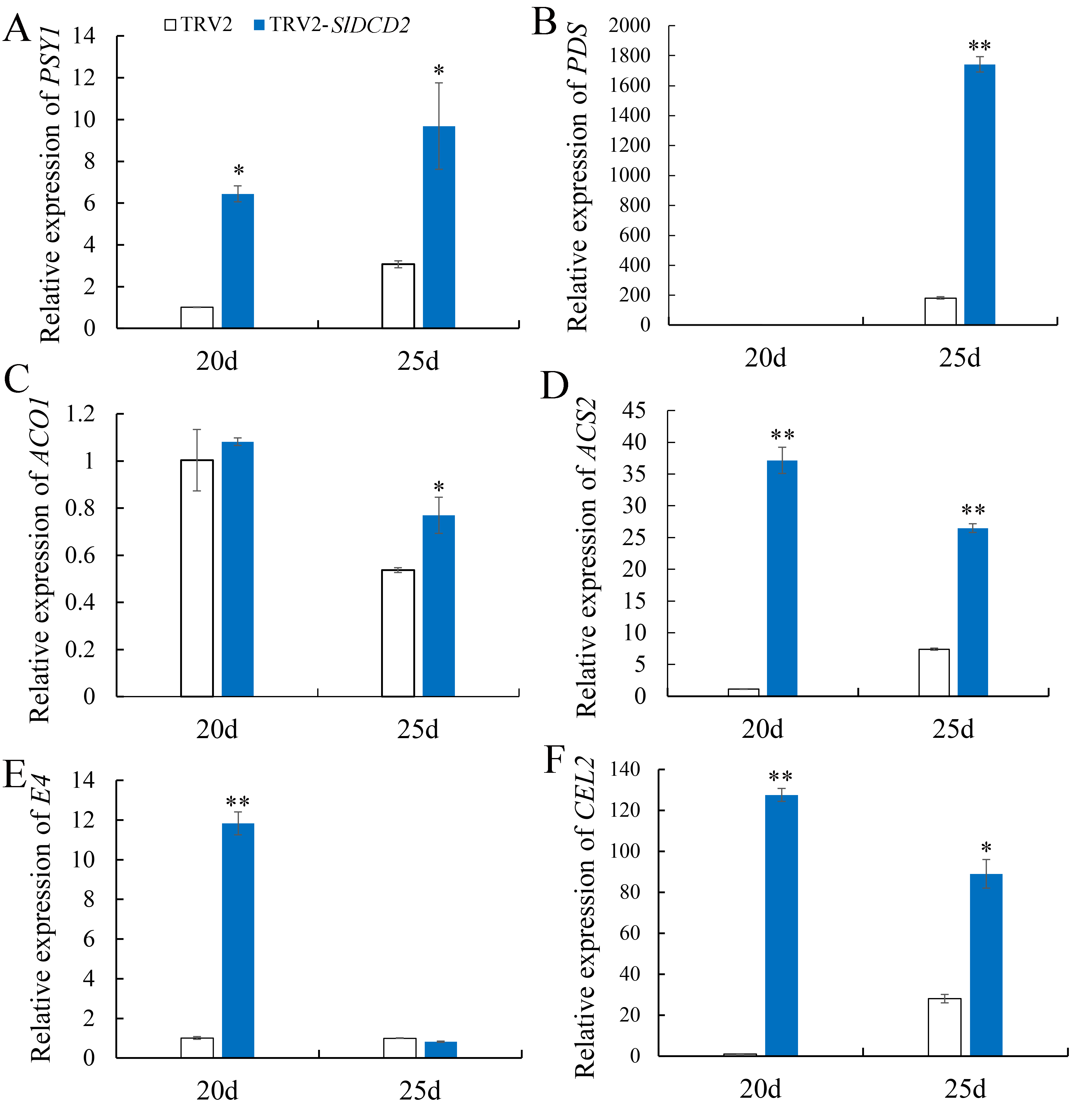


**Figure S2.** The expression of carotenoids biosynthesis genes *PSY1* (A) and *PDS* (B), ethylene synthesis genes *ACO1* (C) and *ACS2* (D), ethylene responsive gene *E4* (E) and cell wall metabolism-related genes *CEL2* (F) in fruits of wild type (WT) and *SlDCD2*-silenced tomatoes for 20 and 25 days after infection with *Agrobacterium tumefaciens*. The data are expressed as the means of three biological replicates ± SDs. The symbols ** and * indicate *P* < 0.01 and *P* < 0.05, respectively.


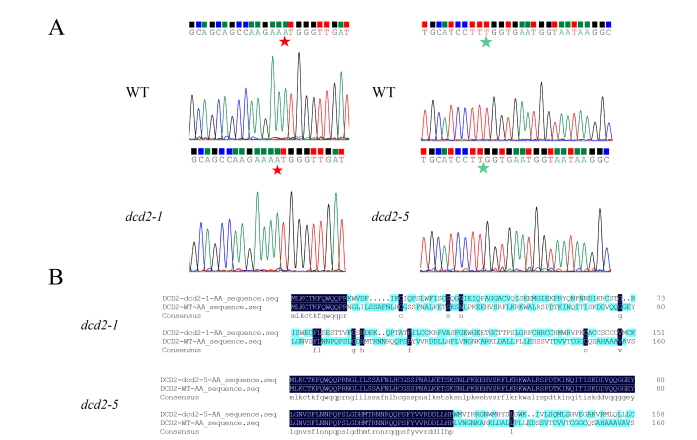


**Figure S3.** Sequencing peak map of *DCD2* CDS sequence and alignment of amino acid sequence near the mutation site in WT and *dcd2* mutants. (A) Sequencing peak map of *DCD2* CDS sequence near the mutation site in WT, *dcd2-1* and *dcd2-5.* The position marked by the red pentagram was the “A” base insertion in the CDS sequence of *DCD2*. The position marked by the green pentagram was the “T” base deletion in the CDS sequence of *DCD2*. (B) Alignment of amino acid sequence near the mutation site in WT, *dcd2-1* and *dcd2-5.*


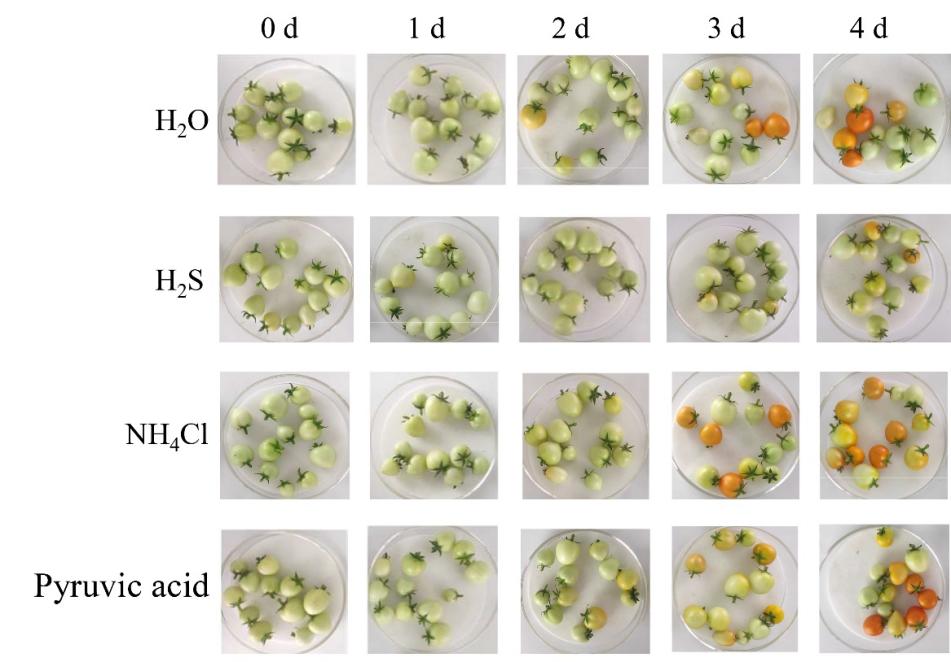


**Figure S4.** Phenotypic of wild-type tomato fruits at white mature stage soaked with distilled water, 50 μM NaHS (H_2_S donor) solution, 50 μM pyruvate solution, 50 μM ammonium chloride solution for 8 h at 0 d, 1 d, 2 d and 3 d after treatment. Water treatment was used as a negative control.

**Table S1.** Primers used in the present study.

| Primers | Sequence | Gene ID | Remarks |
| --- | --- | --- | --- |
| *SlDCD2*-F | GAAAGATTGCTCAAGAAACGGG | Solyc01g008900 | qPCR |
| *SlDCD2*-R | GAGCTAGACCAAACATACCGAG |  |  |
| *SlSGR1*-F | GGCTATCTCCCAAACCATCAA | Solyc08g080090 | qPCR |
| *SlSGR1*-R | ACTCTGCAACAACTTCATCTCT |  |  |
| *SlPAO*-F | CCTCATCGTCTTGCTCCTTTAT | Solyc11g066440 | qPCR |
| *SlPAO*-R | GCAGCTTGAGGTATCCTTGT |  |  |
| *SlNYC1*-F | GATCAGGGAAGAGCACTGTATG | Solyc07g024000 | qPCR |
| *SlNYC1*-R | GAGAACACAGACACCCAAGTAT |  |  |
| *SlPSY1*-F | GAAGATGCCAGAAGAGGAAGAG | Solyc03g031860 | qPCR |
| *SlPSY1*-R | GGTCACCCTTCCAGCAAATA |  |  |
| *SlPDS*-F | CAAGACCAGAGCTGGACAATAC | Solyc03g123760 | qPCR |
| *SlPDS*-R | CAAACCTGCACCAGCAATAAC |  |  |
| *SlE4*-F | CATGACCCGACCACTCTAAATC | Solyc03g111720 | qPCR |
| *SlE4*-R | CAGTTGAGCCTGAGCATCAT |  |  |
| *SlACO1*-F | CCATGTCCTAAGCCCGATTT | NM_001247095.2 | qPCR |
| *SlACO1*-R | GGCCACTCACTTTGTCATCT |  |  |
| *SlACS2*-F | GAGGTTCGTAGGTGTTGAGAAA | Solyc01g095080 | qPCR |
| *SlACS2*-R | GGAGGAATAGGTGACGAAAGTG |  |  |
| *SlCEL2*-F | CTGCTGTTTGCCCTTTCTATTG | Solyc09g010210 | qPCR |
| *SlCEL2*-R | TGCCTTCTTCTTGTTGCTTTATG |  |  |
| *SlEXP*-F | CCGACGATTGGACACCTAAA | Solyc06g051800 | qPCR |
| *SlEXP*-R | GGTCCTCCTTAATCAAAGGACATA |  |  |
| *SlTubulin*-F | TAGAGCCTGGTACGATGGATAG | Solyc08g006890 | qPCR |
| *SlTubulin*-R | CAACTCAGCGCCTTCAGTATAA |  |  |
| *SlSOD*-F | FCAGAGGGTGCTGCTTTACAA | NM_001247840.2 | qPCR |
| *SlSOD*-R | GGTCACAAGAGGGTCCTGAT |  |  |
| *SlCAT*-F | TGATGTTTGTCTCCCAACGG | Solyc12g094620 | qPCR |
| *SlCAT*-R | GTGCTTTCCCCTCTTTGTTAATC |  |  |
| *SlAPX*-F | GCAGCTGCTGAAGGAGAAGT | NM_001247702.2 | qPCR |
| *SlAPX*-R | CACTGGGGCCACTCACTAAT |  |  |
| *SlPOD1*-F | CACAACTCCAATGCAACTGC | Solyc02g079500 | qPCR |
| *SlPOD1*-R | TCCCCGGTCAAAACTTGATC |  |  |
| *SlPOD2*-F | TGTTGATGGTTGTGATGGAGG | Solyc02g079510 | qPCR |
| *SlPOD2*-R | TCACTTCAAAGCCTCTGACTG |  |  |
| *SlDCD2*-TRV2-F | GTGAGTAAGGTTACCGAATTCATGTTGAAATGCACAAAGTTTCAAT | Solyc01g008900 | VIGS |
| *SlDCD2*-TRV2-R | CGTGAGCTCGGTACCGGATCCGAGGGAGTAGTGCATCCAGTTTCC |  |  |
| *SlDCD2*-pCOLD-F | CTCGGTACCCTCGAGGGATCCATGTTGAAATGCACAAAGTTTCAAT | Solyc01g008900 | Recombinant protein expression |
| *SlDCD2*-pCOLD-R | AGCAGAGATTACCTATCTAGATCAGCATATCTCGAAGTAAGATTTGTAA |  |  |
| *SlDCD2*-Cas9-F1 | GTCACAATGGCAGCAGCCAAGAAA | Solyc01g008900 | construct CRISPR/Cas9-*SlDCD2* |
| *SlDCD2*-Cas9-R1 | AAACTTTCTTGGCTGCTGCCATTG |  |  |
| *SlDCD2*-Cas9-F2 | GTCAGGATGCACTACTCCCTCTCT |  |  |
| *SlDCD2*-Cas9-R2 | AAACAGAGAGGGAGTAGTGCATCC |  |  |
| *SlDCD2*-DNA-F1 | TAGAGAAATTTCTGTGGCAATATCAC | Solyc01g008900 | DNA identification of *dcd2* mutants |
| *SlDCD2*-DNA-R1 | CACTGATGTTTGAGACTACAAAGAC |  |  |
| *SlDCD2*-DNA-F2 | GGGAGGTGAATATCTTGGGAA |  |  |
| *SlDCD2*-DNA-R2 | CTTCAATACCATGTTTATGAGTTGCT |  |  |
| *SlDCD2*-CDS-F | ATGTTGAAATGCACAAAGTTTCAAT | Solyc01g008900 | cDNA identification of *dcd2* mutants |
| *SlDCD2*-CDS-R | TCAGCATATCTCGAAGTAAGATTTG |  |  |
